# PERCEIVED FINANCIAL VULNERABILITY, WEALTH, AND WEALTH CHANGE: THE HEALTH AND RETIREMENT STUDY

**DOI:** 10.1093/geroni/igac059.3059

**Published:** 2022-12-20

**Authors:** Madison Maynard, Emily Flores, Daniel Paulson, Peter Lichtenberg

**Affiliations:** University of Central Florida, Orlando, Florida, United States; Wayne State University, Detroit, Michigan, United States; University of Central Florida, Orlando, Florida, United States; Wayne State University, Detroit, Michigan, United States

## Abstract

Financial vulnerability among older adults is an escalating social concern given all too frequent financial exploitation of this population. The 6-item Perceived Financial Vulnerability (PFV) was derived from the 56-item Lichtenberg Financial Decision Rating Scale to examine awareness and psychological vulnerability regarding finances. It was included as an experimental module in the Health and Retirement Study (HRS) in 2018. Prior findings have identified significant associations of PFV with wealth, demographics, and health status. The goals of the current study were to examine the relationship between wealth, changes in wealth, and perceived financial vulnerability. The sample included 1156 respondents to the HRS. Respondents were 57.5% female and 77.2% Caucasian. Average age was 68.28 (SD = 10.74) and average years of education was 13.11 (SD = 2.88). The PFV demonstrated adequate internal consistency (α = .60). Total assets at baseline (2016) and change in total assets over two waves (2016 to 2018) were independently stratified into deciles and used as primary predictors of perceived financial vulnerability in 2018. A multiple linear regression model was conducted to examine these relationships. Consistent with previous findings, demographics (R2=0.04, F(5,1150)=10.07, p < .001) and baseline wealth (B= -0.20, SE=0.02, p < .001) predicted PFV scores. Subsequent addition of wealth change (B=-0.06, SE=0.02, p=.002) significantly contributed to overall variance accounted for (p < .01). Negative wealth change over two years and low baseline wealth related to higher PFV. These findings support the construct and the PFV measure as valid and informative.

